# Stress Hormone Epinephrine Enhances Adipogenesis in Murine Embryonic Stem Cells by Up-Regulating the Neuropeptide Y System

**DOI:** 10.1371/journal.pone.0036609

**Published:** 2012-05-03

**Authors:** Ruijun Han, Joanna B. Kitlinska, William R. Munday, G. Ian Gallicano, Zofia Zukowska

**Affiliations:** 1 Department of Integrative Biology and Physiology, Stress Physiology Center, University of Minnesota, Minneapolis, Minnesota, United States of America; 2 Departments of Physiology & Biophysics, Georgetown University Medical Center, Washington, D.C., United States of America; 3 Departments of Biochemistry & Cellular and Molecular Biology, Georgetown University Medical Center, Washington, D.C., United States of America; Pennington Biomedical Research Center, United States of America

## Abstract

Prenatal stress, psychologically and metabolically, increases the risk of obesity and diabetes in the progeny. However, the mechanisms of the pathogenesis remain unknown. In adult mice, stress activates NPY and its Y2R in a glucocorticoid-dependent manner in the abdominal fat. This increased adipogenesis and angiogenesis, leading to abdominal obesity and metabolic syndrome which were inhibited by intra-fat Y2R inactivation. To determine whether stress elevates NPY system and accelerates adipogenic potential of embryo, here we “stressed" murine embryonic stem cells (mESCs) *in vitro* with epinephrine (EPI) during their adipogenic differentiation. EPI was added during the commitment stage together with insulin, and followed by dexamethasone in the standard adipogenic differentiation medium. Undifferentiated embryonic bodies (EBs) showed no detectable expression of NPY. EPI markedly up-regulated the expression NPY and the Y1R at the commitment stage, followed by increased Y2R mRNA at the late of the commitment stage and the differentiation stage. EPI significantly increased EB cells proliferation and expression of the preadipocyte marker Pref-1 at the commitment stage. EPI also accelerated and amplified adipogenic differentiation detected by increasing the adipocyte markers FABP4 and PPARγ mRNAs and Oil-red O-staining at the end of the differentiation stage. EPI-induced adipogenesis was completely prevented by antagonists of the NPY receptors (Y1R+Y2R+Y5R), indicating that it was mediated by the NPY system in mESC's. Taken together, these data suggest that stress may play an important role in programing ESCs for accelerated adipogenesis by altering the stress induced hormonal regulation of the NPY system.

## Introduction

Obesity is a major problem in the Western world, and an increasing challenge in the developing countries. In particular, abdominal obesity is linked to chronic diseases such as type 2 diabetes, hypertension and cardiovascular disease, currently recognized as major public health concerns [1], [2]. Estimates indicate that obesity is responsible for more than 300,000 deaths each year in the United States alone, triggering the largest growth in mortality over the past decade [3]. Owing to the increase in obesity, life expectancy may start to decrease in developed countries for the first time in the recent history [4].

Adipose tissue is one of the most dynamic tissues of the body, expanding and shrinking in response to various hormonal, neurogenic and nutritional stimuli due to both adipocyte hypertrophy and hyperplasia [5], [6], [7]. The number of adipocytes is set before adolescence, and stays relatively constant throughout the adulthood [7], while adipose stem cells are mainly committed in pregnancy [8]. In humans and rodents, prenatal stress increases the risk of obesity and diabetes in the progeny [9], [10]. However, how stress affects adipogenic commitment in embryos, and contributes to the adult obesity, remains unclear.

Adipogenesis is a developmental process by which mesenchymal stem cells (MSCs) differentiate into mature adipocytes. Terminal stages of adipogenic differentiation have been extensively studied using immortalized preadipocyte lines [11]. However, very little is known regarding the early steps of adipogenesis, in particular, the molecular mechanisms and the cellular intermediates responsible for the transitions from undifferentiated embryonic stem cells (ESCs) to MSCs, and from MSCs to preadipocytes [12]. ESCs, which are derived from the inner cell mass of the blastocyst embryos, have the potential to differentiate into all cell types *in vitro* and *in vivo*, thus representing an ideal study system for regenerative medicine. ESCs can be maintained in an undifferentiated state in the presence of leukaemia inhibitory factor (LIF) [13]. By adding appropriate differentiation agents, ESCs can be committed to a variety of cell types, including adipocytes [14], cardiac cells [15], skeletal-muscle cells [16], neurons [17] and blood cell lineages [18]. Adipocytes arise from mesenchymal stem cells [19], a common precursor for myocytes, chondrocytes and osteocytes. Once committed to the adipocyte lineage, adipocyte progenitors mature into adipocytes during the terminal stages of differentiation [20].

Stress has been linked to the pathogenesis of obesity in adult animals and humans [21], [22]. In our previous mouse study, stress was found to accelerate and amplify the development of high fat diet-induced obesity by up-regulating the release of NPY and the expression of its Y2 receptor (Y2R) within the adipose tissue. The NPY-Y2R system increased fat accretion by directly stimulating differentiation of adipocytes and by indirectly increasing angiogenesis, both via Y2R [22].

NPY is a major sympathetic neurotransmitter activated by stress [23], [24], [25]. Additionally, NPY is also expressed by non-neuronal cells, including platelets/megakaryocytes, immune cells and adipocytes [22], [26]. The peptide has both central and peripheral effects that are important in cardiovascular, neuro-endocrine and metabolic adaptations to environmental and nutritional stress which has the potential to threaten the organism's ability to survive [27]. On the cellular level, NPY has been implicated in promoting growth and/or differentiation of a variety of cells, in a receptor-specific manner. Via its Y1R, NPY stimulates proliferation of neuroprogenitors [28] and osteoblasts [29], whereas via the Y2R/Y5R it promotes angiogenesis and preadipocyte 3T3-L1 differentiation [22]. The latter, the Y2R mediated actions appear to require the presence of dipeptidyl peptidase IV (DPPIV). DPPIV cleaves NPY_1–36_ to a shorter form, NPY_3–36_, which is inactive for the Y1R and selectively activates the Y2R and the Y5R [22]. NPY has also been shown to stimulate proliferation of human embryonic stem cells mediated by the Y1R and Y5R [30].

Stress induces a cascade of adaptive neuroendocrine responses, which classically includes secretion of cortisol and epinephrine (EPI) into the circulation from the adrenal gland [31]. Following sympathetic nerve activation, NPY and norepinephrine (NE) are co-released, activating various physiological processes [32], both synergistically and independently. For example, EPI [33] and glucocorticoids [22] increase NPY expression and signaling [34] in both neuronal and non-neuronal cells. To examine the effects of stress and its mediators in embryonic adipogenesis, we treated mESCs with stress hormone EPI. Here we demonstrate that EPI up-regulates NPY expression, which accelerates and amplifies adipogenic differentiation of mESCs.

## Results

### EPI increased the expression of NPY and its receptors in mESCs

**Figure 1:**
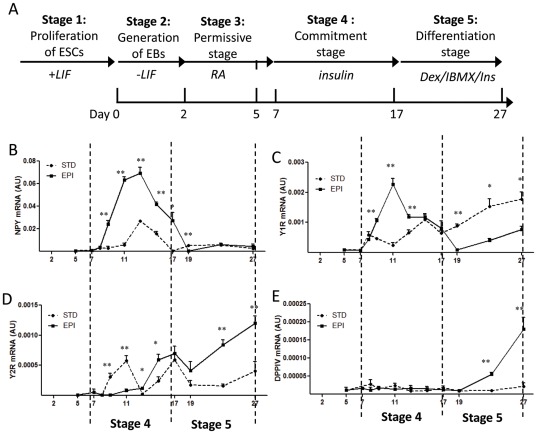
Epinephrine up-regulated the NPY system during mESCs adipogenesis. (A) Schematic representation of the strategy for differentiation of mESCs into adipocytes. (B–G) Gene expression analysis of NPY system during mESCs adipogenesis. EBs were treated with insulin (standard, STD) or insulin plus 1 µM EPI (EPI) from day 7 to day 17 during commitment stage, mRNA levels of NPY (B), Y1R (C), Y2R (D) and DPPIV (E) were measured by real time PCR. β-actin expression was used as an internal control. Values are from three separate experiments each performed in triplicate. *p<0.05, **p<0.01.

We used the five-stage method to direct the differentiation of mESCs into adipocytes (Figure 1A). First, we expanded the undifferentiated mESCs in the presence of LIF (stage 1). When LIF is withdrawn, mESCs spontaneously differentiated into three-dimensional cellular aggregates or embryonic bodies (EBs) in suspension (stage 2). Two days later, the EBs were treated with all-*trans* retinoic acid (RA) for 3 days and entered the permissive stage (stage3). Between day 7 and day 17, a period known as the commitment stage (stage 4), cells were exposed to insulin which induced their commitment to the adipose cell lineage. The final stage (stage 5), between day 17 to day27, is known as the terminal differentiation stage, when the cells are exposed to the classic adipogenic factors: insulin, dexamethasone (Dex) and methylisobutylxanthine (IBMX).

To investigate the role of the stress hormone EPI in the regulation of NPY and its receptors in ESCs, EPI was added from day 7 to day 17 during the commitment stage, and cells were harvested for detection of mRNA expression of NPY, Y1R, Y2R and DPPIV at the different time points. In undifferentiated EBs, NPY was undetectable but became up-regulated during adipogenic differentiation with insulin at the commitment stage (Figure 1B). EPI markedly enhanced the expression of NPY at that stage (Figure 1B). The up-regulation of the expression of NPY was mirrored by the up-regulation of the Y1R mRNA (Figure 1C) at the commitment stage and this was followed by up-regulation of the Y2R at the late of commitment stage and the differentiation stage (Figure 1D). DPPIV mRNA was also upregulated at the differentiation stage (Figure 1E). EPI markedly increased the NPY and Y1R expression at the commitment stage (Figure 1C), which peaked on day 4 of insulin-induced adipogenic commitment (Figure 1C). No difference in DPPIV expression was observed during the commitment stage (Figure 1E).

### EPI augmented and accelerated differentiation of mESCs into adipocytes

**Figure 2:**
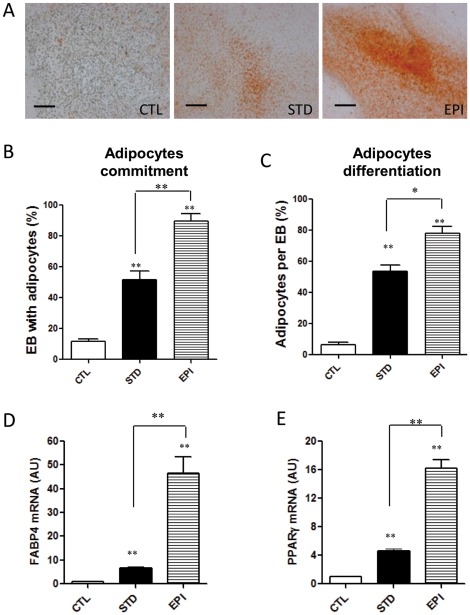
Epinephrine augmented and accelerated the differentiation of mESCs into adipocytes at the end of differentiation. (A) Images of adipocytes developed from EBs treated without insulin at the end of differentiation (day 27) (CTL), with insulin (STD) or insulin plus epinephrine (EPI) added from day 7 to day 17 during commitment stage. Scale bars = 150 µm. (B) Percentage of EB outgrowths with adipocyte colonies and (C) percentage of adipocytes per EB outgrowth were measured and quantified at day 27. (D–E) Gene expression of adipocyte markers was measured by real time PCR at the end of the differentiation (day 27). RNA was isolated from differentiated mESCs and analyzed for expression of adipocyte marker genes FABP4 and PPARγ. β-actin expression was used as an internal control. Values are from three separate experiments each performed in triplicate. *p<0.05, **p<0.01.

At the end of differentiation, we measured mature adipocytes in EB outgrowths by oil red-O staining, a specific stain for triglycerides. mESCs treated with EPI during the commitment stage had more adipocytes compared to standard medium, indicating that EPI-induced stress stimulated adipogenesis (Figure 2A). Treatment with insulin during commitment stage from day 7 to day 17 promoted a high rate of adipogenesis resulting in the formation of adipocyte colonies in 60% of EB outgrowths (Figure 2B). Co-treatment with EPI during this stage significantly increased adipogenesis, resulting in more than 90% of the EB outgrowths forming adipocytes (Figure 2B). This suggested that EPI induced adipocytes commitment in mESCs [35]. Co-treatment with EPI during this stage also increased the percentage of adipocytes per EB outgrowth, suggesting that EPI also promote the terminal differentiation (Figure 2C) [12]. EPI markedly upregulated the expression of the adipocyte markers FABP4 and PPARγ at the end of differentiation (Figure 2D–E) in parallel to increase of the Y2R and DPPIV mRNAs at this stage, compared to standard control (Figure 1D–E).

### EPI-induced adipogenesis is mediated by the NPY system

**Figure 3:**
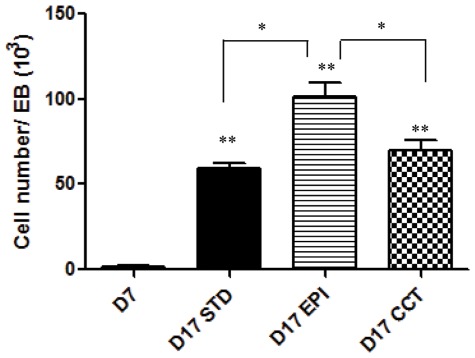
The NPY receptors antagonists blocked epinephrine-induced proliferation of EB cells. Four EBs per well were plated on gelatin-coated 12-well plates at day 7 and treated with insulin (STD), insulin plus epinephrine (EPI), insulin plus EPI and NPY receptors (Y1R+Y2R+Y5R) antagonists (CCT). The number of cells was measured at day 7 and day 17. Values are from three separate experiments each done in ten wells. *p<0.05, **p<0.01.

**Figure 4:**
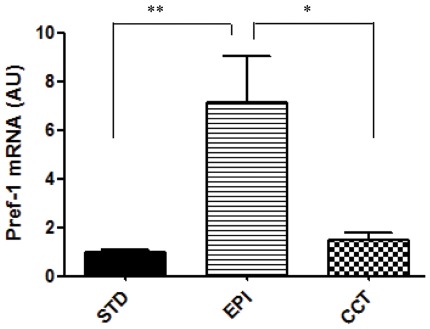
The NPY receptors antagonists prevented the effects of epinephrine on up-regulation of the preadipocyte marker Pref-1 at commitment stage. EBs were treated with insulin (STD), insulin plus epinephrine (EPI) or insulin plus EPI and NPY receptors antagonists (CCT) from day 7 to day 17. RNA was isolated after 10days of treatment and the expression of preadipocyte marker Pref-1 was measured by RT-PCR. β-actin expression was used as an internal control. Values are from three separate experiments each performed in triplicate*p<0.05, **p<0.01.

EPI significantly increased EB cells proliferation at the commitment stage (Figure 3). Consistent with the EPI-induced upregulation of percentage of EB outgrowths with adipocyte colonies (Figure 2B), EPI markedly increased the expression of the preadipocyte marker Pref-1 at the commitment stage, (Figure 4), indicating that EPI induces more cells committed to preadipocytes stage.

**Figure 5:**
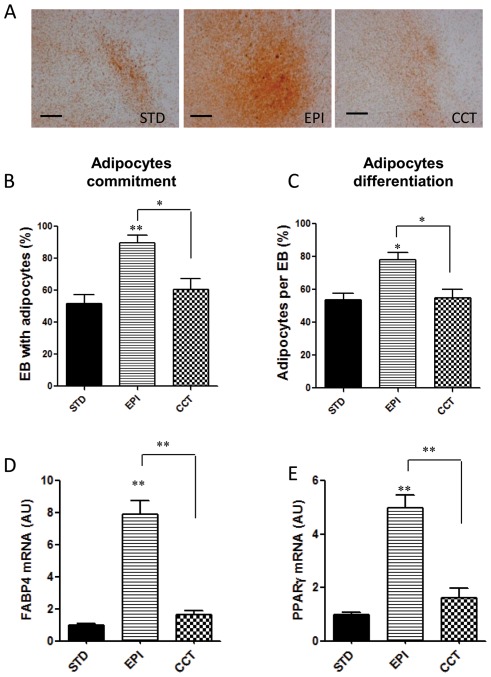
Epinephrine-induced adipogenesis is mediated by the NPY system. (A) EBs were treated with insulin (STD), insulin plus epinephrine (EPI) or insulin plus EPI and NPY receptors antagonists (CCT) from day 7 to day 17.Final differentiated adipocytes were detected by oil red O-staining. Scale bars = 150 µm. (B) Percentage of EB outgrowths with adipocyte colonies and (C) percentage of adipocytes per EB outgrowth were measured and quantified at day 27. (D–E) mRNA levels of adipocyte markers were measured by RT-PCR at the end of the differentiation. RNA was isolated from differentiated mESCs at day 27, and analyzed for expression of adipocyte marker genes FABP4 (D) and PPARγ (E). β-actin expression was used as an internal control. Values are from three separate experiments each perfoemed in triplicate *p<0.05, **p<0.01.

To explore whether the effects of EPI-induced mESCs adipogenesis were mediated by the NPY system, antagonists of the NPY receptors (Y1R+Y2R+Y5R) were added during the commitment stage together with EPI. Antagonists of the NPY receptors completely blocked the effects of EPI on up-regulation of the expression of the preadipocyte marker Pref-1 (Figure 4) during the commitment stage. The EPI-induced increase in EB cells proliferation was also blocked by antagonists of the NPY receptors (Figure 3). Finally, antagonists of the NPY receptors (Y1R+Y2R+Y5R) completely prevented the effects of EPI-induced stress on adipogenic differentiation, measured by oil red-O staining and expression of adipocyte markers at the end of differentiation (Figure 5). Antagonists of the NPY receptors also blocked the effects of EPI on increase of percentage of EB outgrowths with adipocytes and percentage of adipocytes per outgrowths (Figure 5B–C).

## Discussion

Stress has been linked to obesity via multiple mechanisms: centrally, through its hypothalamic actions on food intake, and peripherally, via direct actions on adipose tissue of sympathetic neurotransmitters, NPY [22] and catecholamines [36], parasympathetic activity [37] and glucocorticoids [38]. Activation of EPI is thought to be critical in the prevention of obesity due to its ability to induce lipolysis in the white fat [36], and thermogenesis in the brown fat [39]. EPI regulates lipolysis in human adipocytes through stimulatory β1- and β2-adrenoreceptors or inhibitory α2-adrenoreceptors [36]. However, the roles of EPI on ESCs differentiation to adipocytes have not been investigated. Here we report that EPI markedly enhanced and accelerated adipogenesis by activating the NPY system in mESCs. We found that EPI stimulated EB cells proliferation (Figure 3) and their commitment to preadipocytes (Figure 4). EPI increased the expression of the preadipocyte marker Pref-1(Figure 4). Pref-1 is specifically expressed in preadipocytes but not in adipocytes and it is used as a preadipocyte marker for MSCs commitment to the adiogenic lineage [8], [40], [41]. EPI-induced up-regulation of Pref-1 indicates that more preadipocytes were committed at this stage, which is consistent with that EPI-induced upregulation of the percentage of EB outgrowths with adipocyte colonies (Figure 2B). Others [42] reported similar effects for thyroid-stimulating hormone (TSH), the main regulator of thyroid growth and function, which has been implicated in the induction of lipolysis in adult animals and was shown to stimulate adipogenesis in mESCs.

Catecholmines, such as EPI in the adrenal medulla and NE in the sympathetic nerves, are important mediators of physiological processes during stress. NPY is co-released with both of them [31], [32], providing a rationale for a functional interaction between catecholamines and NPY. The co-transmission between NPY and EPI has already been demonstrated in the cardiovascular system, where both EPI-induced vasoconstriction and NE-mediated growth of cardiomyocytes are potentiated by co-administration of NPY [43]. In immune cells, EPI-induced inhibition of IL-6 release from macrophages in ex vivo superfused spleen slices is significantly modulated by NPY via its Y1R [44]. Furthermore, EPI-induced leukocytosis is mediated by Y1R and Y5R during leukocyte mobilization [44]. In the present study, EPI markedly up-regulated NPY and stimulated adipogenesis mediated by NPY receptors in mESCs (Figure 5). Antagonists of the NPY receptors (Y1R+Y2R+Y5R) completely blocked the effects of EPI on stem cell proliferation and differentiation (Figure 3, 5). However, adding Y1R antagonist, Y2R antagonist or Y5R antagonist separately during the commitment stage together with epinephrine from day 7 to 17; none of them alone efficiently blocked the EPI-induced adipogenic effects (data not shown).

Understanding the physiological role of NPY is challenging, since up to now five NPY receptors have been cloned, Y1R, Y2R,Y4R, Y5R and Y6R [45], representing G-coupled receptors. Of those, only theY1R requires the whole NPY_1–36_ molecule for its activation. The Y1R has been show to stimulate proliferation of many cells [46], including vascular smooth muscle [47] and human embryonic stem cells [30]. In contrast, the shorter form NPY_3–36_, a product of DPPIV cleavage, while inactive for the Y1R, activates the Y2R and the Y5R, stimulating the proliferation of endothelial cells as well as adipogenesis of preadipocytes [22]. TheY2R and increased levels of NPY in the adipose tissue mediated the accelerated fat accretion induced by chronic stress in adult male mice fed high fat diets [22], leading to abdominal obesity and metabolic-like syndrome. All of these effects of stress were completely prevented by intra-fat Y2R inactivation [22]. While other groups have also reported that the Y1R plays a role in adipogenesis by increasing preadipocyte proliferation [48], the role of the Y5R in adipogenesis remains to be examined further.

In the current study, we hypothesized that NPY and its Y1R and Y2R are sequentially activated and play an important role in the regulation of embryonic stem cell differentiation to adipocytes, particularly under conditions of stress. We propose that the Y1R and Y2R have complementary effects on adipogenesis, where activation of the Y1R leads to proliferation while activation of theY2R promotes adipogenic differentiation. This notion was based on recent findings in 3T3-L1 cells, where the Y1R is highly expressed in preadipocytes and markedly decreased in matured adipocytes. On the other hand, the expression of the Y2R is low in preadipocytes and significantly increased in mature adipocytes (data not shown). Similar opposite patterns of the expression of the Y1R and theY2R was found in the current study in the ESCs, where EPI markedly increased the Y1R expression at the commitment stage while up-regulating the expression of the Y2R and DPPIV at the differentiation stage. The high expression of the Y2R and DPPIV in the later adipogenic stage is consistent with our previous data in vivo showing that stress-induced up-regulation and activation of the Y2R and DPPIV mRNAs in the visceral fat is associated with rapid development of abdominal obesity and metabolic syndrome in adult mice [22].

How the NPY expression is regulated in cells during adipogenic differentiation is not well understood. Epigenetic regulators, such as histone modification and DNA methylation, have been implicated in influencing ESCs self-renewal and differentiation [49]. The NPY gene is rich in CpG islands and is highly hyper-methylated in most of available human embryonic stem cell lines [50]. NPY is suggested to be one of only 25 genes whose silencing is required for totipotency of these cells. In contrast, NPY gene is hypo-methylated in somatic tissues and many differentiated cells [50]. In our pilot studies, we assessed DNA methylation at 3 sites of the NPY promoter, including calmodulin-responsive element (CaM-RE), nerve growth factor responsive element (NGF-RE), and cAMP-RE, using methylation specific real time PCR (data not shown). In undifferentiated murine embryonic bodies, these regions showed high level of DNA methylation and NPY mRNA was undetectable. However, EPI induced up-regulation of NPY expression associated with significant decrease in DNA methylation at the NGF-RE and CaM-RE sites (data not shown). Previous studies have already demonstrated that NGF, glucocorticoids and EPI are strong promoters of NPY expression in many cells [22], [33], [34], [51]. Stimulation of the calmodulin pathway was also reported with increased adipogenesis of the ESCs [52]. Therefore, our observations shed new light on mechanisms by which stress may induce epigenetic changes in the NPY system, and thus stimulate future adipogenesis during embryogenesis.

A large numbers of epidemiological and animal studies have demonstrated prenatal stress has long term, programming effects on obesity and metabolic syndrome [53], [54]. Here we show that the stress hormone EPI stimulates ESC adipogenesis mediated by the NPY system. Our data suggested that NPY system mediates stress induced adipogenic commitment in embryo, and thus play an important role in prenatal stress programmed abdominal obesity and metabolic syndrome in offspring.

## Materials and Methods

### Materials

NPY was obtained from Bachem (San Carlos, CA), Y1R antagonist, BIBP 3226 from Sigma-Aldrich (St. Louis, MO); Y2R antagonist, BIIE 0246 formate and Y5R antagonist, CGP71683, from Tocris (Ellisville, MO).

### Cell culture

The CCE mESC line was a generous gift from Dr. Ian Gallicano (Georgetown University, Washington DC) [55]. Murine ESC were grown in gelatin-coated flasks and maintained in Dulbecco's Modified Eagle's Medium (DMEM) with 4.5 g/l d-glucose, supplemented with 15% (vol/vol) fetal bovine serum (FBS, Stemcell Technologies), 100 U/ml penicillin, 100 µg/ml streptomycin, 0.1 mM non-essential amino acids, 10 ng/ml murine recombinant leukemia inhibitory factor (LIF; Stemcell Technologies), 0.1 mM monothioglycerol, and 2 mM L-glutamine and 1 mM sodium pyruvate (Invitrogen). Adipogenesis was induced as described previously in detail [56] with minor modifications. On day 0, cells were trypsinized and resuspended at 10^5^ cells/ml in the same media as above without added LIF. Drops (10 µl) were plated on the bottom of a 10 cm petri dish (Fisher), and plates were cultured upside-down. On day 2, plates were flipped and the embryonic bodies (EBs) were flooded with media containing 10^−7^ M all-*trans* retinoic acid (Sigma-Aldrich). This media was changed daily to prevent EBs from adhering to the dish. On day 5, media without retinoic acid was added, and on day 7, EBs were collected and plated on gelatin-coated six-well plates. At this point, 5 µg/ml insulin (Sigma-Aldrich) was added to the media for 10 days. On day 17, media was replaced media containing with dexamethasone (Dex) (400 ng/ml; Sigma-Aldrich) and methylisobutylxanthine (IBMX) (500 nM; Sigma). On day 19, DEX and IBMX were removed from the media, and the cells were cultured for 10 additional days in the insulin-containing media to achieve fully differentiate adipocytes. Cells were harvested at indicated times (Figure 1). EPI (1 µM) was were added daily, between day 7 to day 17 with or without the antagonists of the NPY receptor (Y1R+Y2R+Y5R) at the concentration of 0.1 µM each.

### Reverse transcription and Real-time PCR

RNA was isolated using Roche RNA isolation kit and cDNA synthesized by iScriptcDNA synthesis kit (BioRad). Real-time PCR was performed using the ICycleriQ Detection System (BioRad) and theTaqMan PCR Reagent Kit with pre-designed primers and fluorescently labeled probes with the duplicates. The primers were from the Applied Biosystems: β- actin, 16403392; NPY, 16403380; Y1R,16403381; Y2R,14381272; DDPIV,16403393; FABP4, Mm00445880; PPARγ, Mm01184322; Pref-1, Mm00494477. The mRNA expression levels were calculated using the comparative C_T_ method with β-actin as the endogenous reference gene, in accordance withthe Applied Biosystems' ABI PRISM, as described previously [22].

### Oil red O staining

At the end of adipogenic differentiation, mESCs cells were fixed in 4% Paraformaldehyde (PFA, USB Corporation) for 8 min, stained with Oil Red O for 30 min at room temperature and imaged (Nikon, Melville, NY).

### Statistical analysis

The Kruskal-Wallis test for non-parametric measures was applied to compare between treatment groups; p<0.05 was considered statistically significant. Man Whitney U non-parametric test was used to analyze the effects of a single treatment compared to control.
